# Child school injury in Lebanon: A study to assess injury incidence, severity and risk factors

**DOI:** 10.1371/journal.pone.0233465

**Published:** 2020-06-12

**Authors:** Samar Al-Hajj, Ricardo Nehme, Firas Hatoum, Alex Zheng, Ian Pike

**Affiliations:** 1 Health Management and Policy Department, American University of Beirut, Riad El-Solh, Beirut, Lebanon; 2 Department of Biology, American University of Beirut, Beirut, Lebanon; 3 BC Injury Research and Prevention Unit, BC Children’s Hospital Research Institute, Vancouver, British Columbia, Canada; 4 Department of Pediatrics, Faculty of Medicine, University of British Columbia, Vancouver, British Columbia, Canada; University of Florida, UNITED STATES

## Abstract

**Background:**

School-based injuries represent a sizeable portion of child injuries. This study investigated the rates of school-based injuries in Lebanon, examining injury mechanisms, outcomes and associated risk factors.

**Methods:**

Data were prospectively collected by intern school nurses at 11 private schools for the 2018–2019 academic year. Descriptive and inferential analyses were performed. Chi-square comparisons were conducted to determine the significance of any differences in injury rates between boys and girls for each category of school.

**Results:**

4,619 injury cases were collected. The yearly rate for school injuries was 419.1 per 1,000 children for the year 2018–2019. Boys demonstrated a significantly higher injury rate for all mechanisms of injuries, with the exception of being injured while walking, injured in the gym/sports areas, and other areas outside the playground and classroom. Elementary school children had the highest rate of injuries, nearly 2.4 times higher than kindergarten, 2.8 times higher than middle school, and 14.5 times higher than high school. Injuries to the face, upper extremities, and lower extremities were nearly 3 times more common than injuries to other areas of the body. Bumps/hits and bruises were most common—almost 3 times more likely than all other injury types. Injuries were mainly minor or moderate in severity—severe injuries were about 10 times less likely. Most injuries were unintentional, with rates nearly 5 times higher than those with unclear intent and 12 times higher than intentional injuries.

**Conclusions:**

School injuries represent a relatively common problem. Compliance with playground safety standards coupled with the implementation of injury prevention strategies and active supervision at schools can curtail child injuries and ensure a safe and injury-free school environment.

## Background

Injury accounts for a large share of the global burden of disease [1]. Child injury, in particular, is a substantial public health problem that claims more children’s lives than any major diseases combined [1]. Play- and sport-related injury is one of the leading causes of child injury worldwide [2–4]. Most of these injuries occur at school premises where children spend a considerable number of days per year. School-related injury is a major contributor to paediatric-related emergency department visits and hospital admissions; where the majority of the reported injuries were bruises, sprain/strains, and open wounds to the upper extremities, lower extremities and head, overwhelmingly sustained by boys, and mostly occurring during organized sports and recreational activities [5–10]. Injury studies conducted in the United States and Canada confirmed that school injuries accounted for almost 10 to 20% of the overall burden of child injury, costing billions of dollars in hospitalizations and other estimated health care expenditures [3, 6, 9, 10, 11].

An injury refers to the bodily harm sustained as the by-product of an external force and transfer of energy beyond an individual’s physiological tolerance resulting from an unsafe environment, conditions or behaviors [12]. Injuries can happen unintentionally, such as fall, drowning and burn, or intentionally, such as a purposeful assault by one individual upon another. In addition to the inflicted physical and psychological harms, child injuries impose financial repercussions on injured children and their families incurred due to healthcare expenditures, particularly in low- and middle-income countries where universal health coverage is most often lacking.

Limited research has explored child injuries in the Eastern Mediterranean Region, and few have examined the nature, extent and risk factors of school-based injuries in particular [13–22]. Among regional studies, the most commonly reported school injury type was a fracture, with the most prevalent injury cause being fall, sustained mainly by boys of younger age. Bullying, fights and physical attacks were also experienced by school children regionally [13–22]. In Lebanon, school-based injuries have received less attention in the injury research literature. A limited number of studies have focused on examining ‘bullying’ among school children and its impact on the child’s health and academic performance, while other studies reported physical, emotional and verbal abuse inflicted by teachers upon school children [23–25].

The education system in Lebanon follows the American or French system and includes grades 1 to 12 for children 5 to 18 years of age. Kindergarten years, not compulsory, begin before grade 1 and accommodate children ages 3 to 5 years, while elementary school encompasses grade 1 to grade 5, middle school grade 6 to grade 9, and high school grade 10 to grade 12. A typical academic year begins in mid-September or early October and ends in mid-June, for an average of 8.5 months of schooling per academic year. There are approximately 1,500 schools in Lebanon (58% public and 42% private), with a school attendance level of nearly 95 percent [26]. Private schools require payment of tuition fees, while public schools are free of charge, and are supported mainly by government funds and attended by less privileged families.

To the best of our knowledge, no previous studies have estimated injuries occurring at school premises in Lebanon. This underscores the importance of exploring physical injuries sustained by school children, and understanding their mechanisms, extent, and more importantly, their outcomes. The primary objective of this study was to assess the incidence of school-based physical injuries, to identify their characteristics, associated child behaviors, injury severity and potential contributing risk factors, based on analyzing school nurse reported incidents. Understanding the etiology of child school injuries is critical to help inform the design and development of school safety protocols and child injury prevention strategies and awareness programs at schools.

## Methods

### Participants

This cross-sectional prospective study collected injury data on school-based injuries throughout Lebanon. Within the context of this study, we focused on physical injuries, both intentional and unintentional, sustained by all school-age children and occurring within the premises of participating private schools.

In Lebanon, only private schools have sufficient resources to retain intern nurses who were available to complete injury incident report forms and collect data for this study. Therefore, only private schools with a nurse were invited to participate. Public schools and schools without an intern school nurse were excluded. A list of 74 private schools with nurses across various governorates in Lebanon was prepared. Each school was contacted, invited to participate and provided with an official letter of invitation directed to the school administration, explaining the objective and requirements of the study. Eleven schools agreed, representing a convenience sample of 14.8% of the total 74 schools. Across the 11 schools, a total of 74 school months of child injury data was gathered.

### Instrument

A school injury surveillance tool was adapted from a validated Canadian school incident reporting system and was used to capture data at the participating schools. The surveillance tool was reviewed and contextualized with the assistance of school nurses from the Order of Nurses in Lebanon. To ensure validity and reliability, the tool was pilot-tested for one-year at 3 private schools located in the capital city, Beirut, during the academic year of September 2017 to June 2018. Lessons learned were documented and further refinement and modification were integrated into the tool. The final version was adopted in the current study, which was carried out during the academic year of September 2018 to June 2019.

### Procedure

Once finalized, the incident data form—originally in English—was translated into French and Arabic to reflect the official language policy at each school, and to facilitate a rapid and efficient data collection process. The injury surveillance tool captured detailed information related to child school injuries, namely injury type, injury date, injury time and location, body part injured, injury intent, activity at the time of injury, contributing factors and injury severity (classified as minor, moderate or severe). The tool also collected child demographic information including child age, sex and grade level.

A soft copy of the injury surveillance tool was shared with each participating school nurse. Subsequent one-on-one training and monthly follow-up were carried out with school nurses to ensure a robust and efficient data collection process. All injuries that occurred at school premises were documented at the time the injury occurred. Collected data were reviewed to eliminate the possibility of any duplicate records. Cases of injury at home were excluded, and child identity information and personal details were concealed.

### Human subjects approval statement

The study was approved by the American University of Beirut–Institutional Review Board (SBS-2018-0569). The study was also approved by each participating school administration.

### Data analysis

Data collected from all schools were translated into one unified language (English), cleaned and compiled into one database. Data were analyzed using STATA, and used to assess injury incidence and to examine injury characteristics, identify keys trends and at-risk populations. Rates were calculated as the number of injuries per 1,000 students per month, to account for some schools having shorter academic years than others. Rates, with 95% confidence intervals, were calculated for boys and girls, for school grade, nature of the injury, intentionality, injury mechanism, injury severity, injury location, and injury type. Chi-square comparisons were conducted to determine the significance of any differences in injury rates between boys and girls for each grade. Due to the number of comparisons conducted, differences in injury rates were considered significant if p < 0.001.

## Results

A total of 11 schools participated in this study, with a cumulative total of 4,619 school-based injuries reported during the study period. Across all schools, the incidence rate of injuries was 49.3 injuries per 1,000 students per month, which equates to 419.1 for an average 8.5-month academic year. An overwhelming number of boys reported a high injury rate of 61.4 per 1,000 students per month (95% CI 52.6, 70.2) compared to girls with a rate of 37.1 per 1,000 students per month (95% CI 28.3, 46.0), regardless of their ages. The study revealed that the injury rate was highest among elementary students (94.1 per 1,000 students per month, 95% CI 85.5, 102.7) and lowest among high school students (6.2 per 1,000 students per month, 95% CI 4.8, 7.5) Table 1.

**Table 1:** Characteristics of school injuries: Distribution of rates of injury intention, severity and location between gender, 2018–2019.

	Frequency (%)			Rate (per 1,000 students per month) (95% CI)			Gender Comparison
Variable	Boys	Girls	Total	Boys	Girls	Overall	p-value
**Overall**	2866 (62.0)	1753 (38.0)	4619 (100.0)	61.4 (52.6, 70.2)	37.1 (28.3, 46.0)	49.3 (43.1, 55.5)	<0.0001
**School (Grade)**							
Kindergarten (KG 1–3)	492 (60.4)	322 (39.6)	814 (17.6)	48.1 (40.1, 56.1)	31.5 (25.0, 38.0)	39.8 (32.5, 47.1)	<0.0001
Elementary (Grade 1–5)	1899 (61.8)	1173 (38.2)	3072 (66.5)	116.9 (107.4, 126.4)	71.5 (63.9, 79.1)	94.1 (85.5, 102.7)	<0.0001
Middle (Grade 6–9)	386 (64.8)	210 (35.2)	596 (12.9)	41.2 (33.6, 48.8)	20.4 (15.0, 25.8)	30.3 (23.8, 36.8)	<0.0001
High (Grade 10–12)	85 (65.9)	44 (34.1)	129 (2.8)	7.9 (6.4, 9.5)	4.3 (3.2, 5.5)	6.2 (4.8, 7.5)	<0.0001
Unknown/Missing			8 (0.2)			0.1 (0.0, 0.2)	
**Injury Location**							
Playground	2041 (63.9)	1151 (36.1)	3192 (69.1)	43.8 (40.2, 47.4)	24.4 (21.8, 27.1)	34.1 (30.9, 37.2)	<0.0001
Classroom	279 (62.3)	169 (37.3)	448 (9.7)	6.0 (4.6, 7.3)	3.6 (2.5, 4.6)	4.8 (3.6, 6.0)	<0.0001
Gym/Sports area	119 (49.2)	123 (50.8)	242 (5.2)	2.6 (1.7, 3.4)	2.6 (1.7, 3.5)	2.6 (1.9, 3.3)	0.8694
Other	80 (50.3)	79 (49.7)	159 (3.4)	1.7 (1.0, 2.4)	1.7 (1.0, 2.4)	1.7 (1.2, 2.2)	0.8795
Unknown/Missing			578 (12.5)			6.2 (4.8, 7.5)	
**Intention**							
Unintentional	1612 (62.5)	966 (37.5)	2578 (55.8)	34.6 (31.4, 37.8)	20.5 (18.0, 23.0)	27.5 (24.7, 30.4)	<0.0001
Intentional	131 (72.8)	49 (27.2)	180 (3.9)	2.8 (1.9, 3.7)	1.0 (0.5, 1.6)	1.9 (1.2, 2.7)	<0.0001
Unclear	327 (63.9)	185 (36.1)	512 (11.1)	7.0 (5.6, 8.5)	3.9 (2.8, 5.0)	5.5 (4.2, 6.7)	<0.0001
Unknown/Missing			1349 (29.2)			14.4 (12.3, 16.5)	

School-related injuries were mostly unintentional with a rate of 27.5 (95% CI 24.7, 30.4), while 1.9 (95% CI 1.2, 2.7) were intentional, and 5.4 (95% CI 4.2, 6.7) had no clear intent. The rates of unintentional injuries were about 5 times higher than unclear intent and 14 times higher than intentional injuries. Further analysis showed an association between child sex and injury intent. Boys were responsible for significantly more intentional injuries than girls (p<0.0001) with injury rates of 2.8 (95% CI 1.9, 3.7) and 1.0 (95% CI 0.5, 1.6) respectively, and reported being involved in physical aggression and fights more often than their female counterparts.

The most common nature of injury involved lower extremity injuries (14.2, 95% CI 12.2, 16.4), face (14.2, 95% CI 12.2, 16.3), and upper extremities (12.5, 95% CI 10.6, 14.4). Head injuries (4.4, 95% CI 3.2, 5.5) were the fourth most prevalent injury among school children (n = 409), of which 132 were moderate injuries and 3 were severe. Compared to girls, boys were significantly more likely to sustain injuries to all areas of the body (p<0.0001).

Various locations within schools contributed to different types and rates of injuries. The most commonplace for occurring injuries was the playground with a rate of 34.1 per 1,000 students per month (95% CI 30.9, 37.2), followed by classrooms with a rate of 4.8 (95% CI 3.6, 6.0). For these two locations, boys had significantly higher injury rates than girls (p<0.0001), but for all other locations, there were no significant differences in injury rates between the genders.

**Fig 1:**
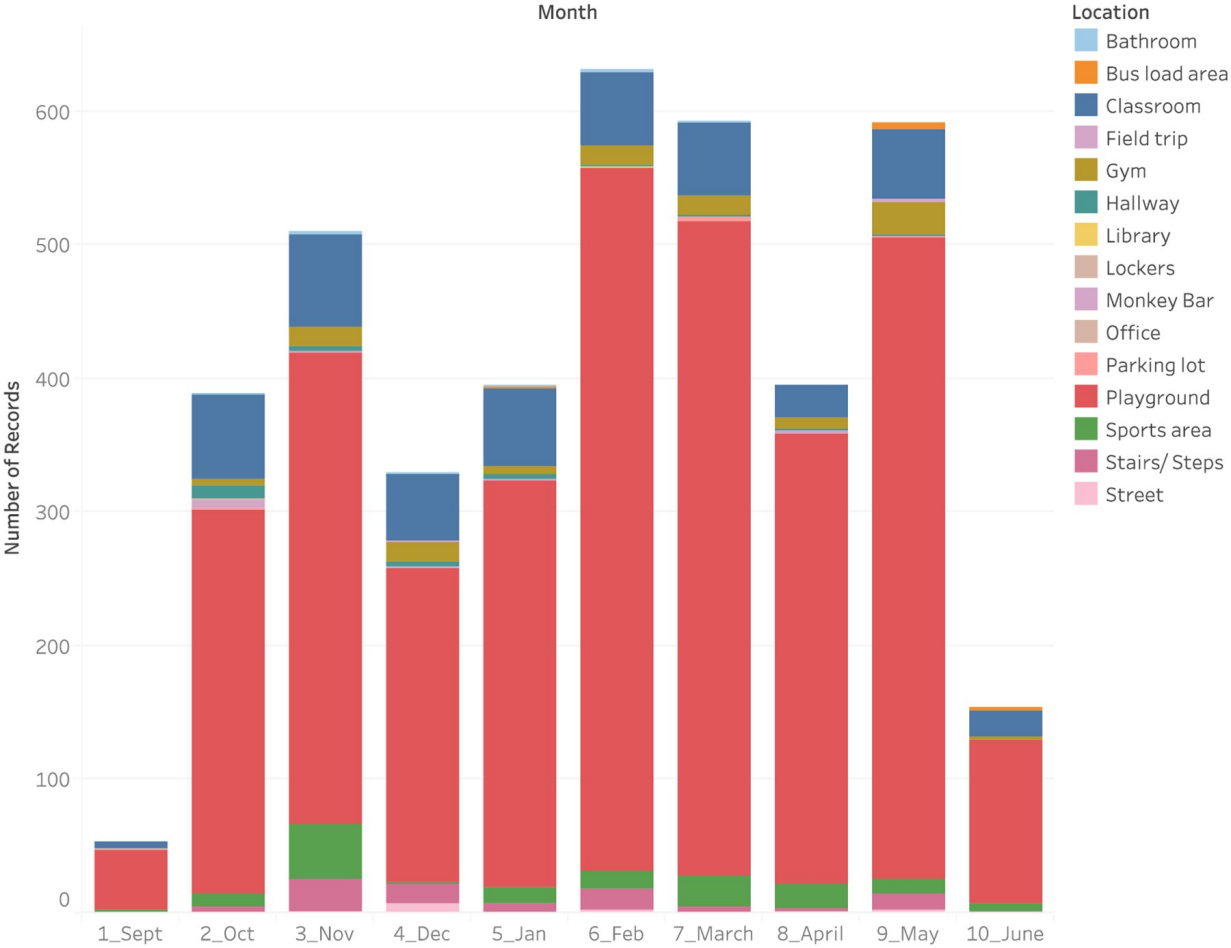
Seasonal distribution of injuries by school location (Sept-June).

Seasonality was a predictor of injury. The analysis revealed a positive association between the injury nature and the warm season. The highest proportion of injuries occurred during February (12.8%), March (15.3%) and May (16.7%)—the month of April coincides with school closure during the Easter vacation (Fig 1).

Playing, running, and walking were the most common mechanism of injury, with injury rates of 24.2 (95% CI 21.5, 26.9), 9.9 (95% CI 8.2, 11.6), and 2.7 (95% CI 1.8, 3.6) per 1,000 students per month, respectively. Boys had higher rates of injury while playing and running when compared with girls (p<0.0001), however, there were no significant differences between boys and girls for injuries sustained while walking.

Most school children reported mild to moderate injuries. The rate of minor injuries was 20.1 (95% CI 17.7, 22.5) as compared to moderate injuries (17.2, 95% CI 15.0, 19.5), which required basic first aid 45.9% of the time. Twelve cases of injury were severe (0.2, 95% CI 0.0, 0.4), necessitating medical attention at local hospitals Emergency Departments. Compared with girls, boys had a significantly higher injury rate for all three levels of injury severity (p<0.0001).

**Fig 2:**
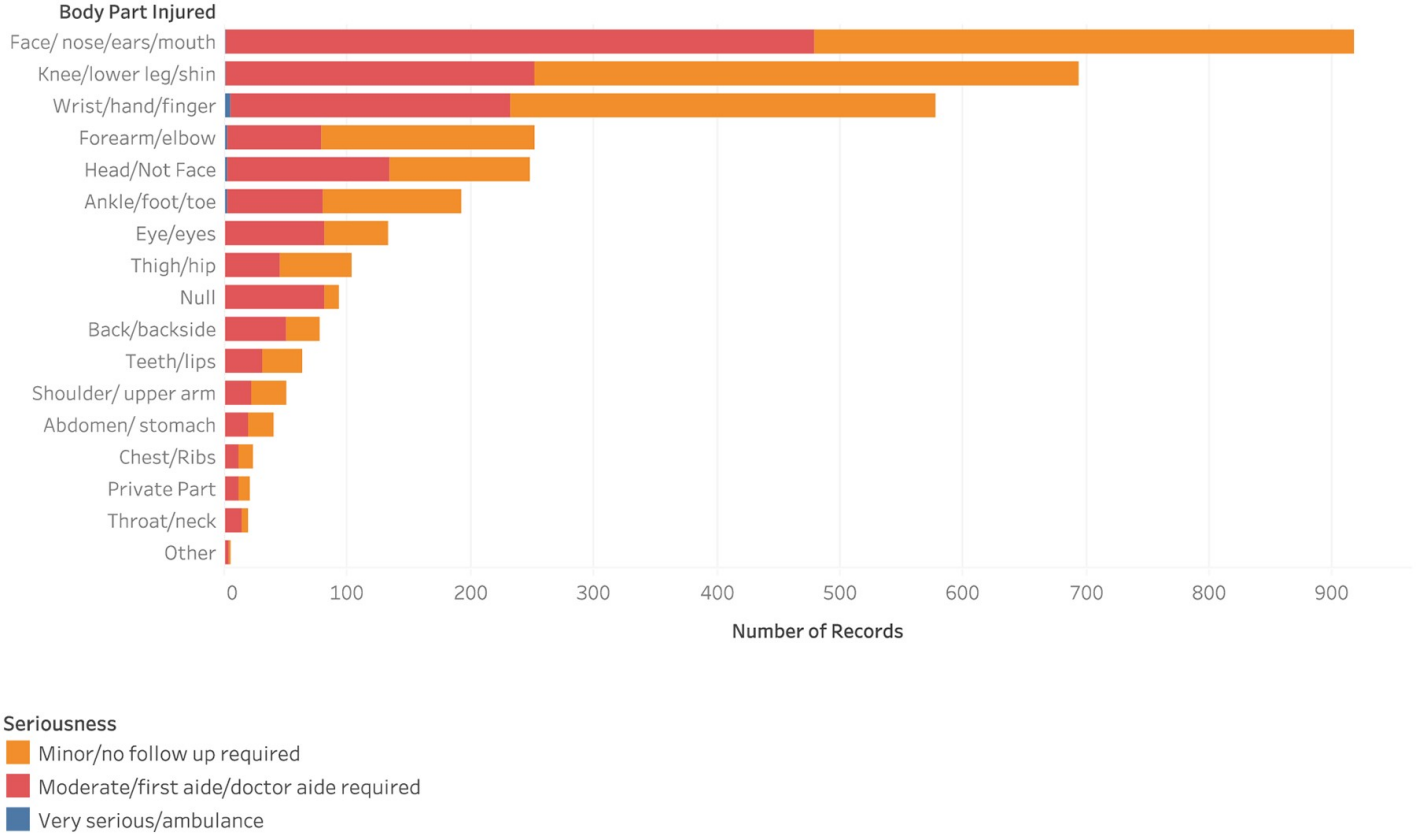
Body parts affected by injuries and corresponding severity.

The response following the incident mainly depended on the seriousness of the injury. The most common response provided by the school nurse was to administer on-site care, which included the application of a ‘band-aide/bandage/ice’ in 64.6% of the cases, and ‘cleaned/washed/isolated wound/area’ in 26.9% of the cases (Fig 2).

**Fig 3:**
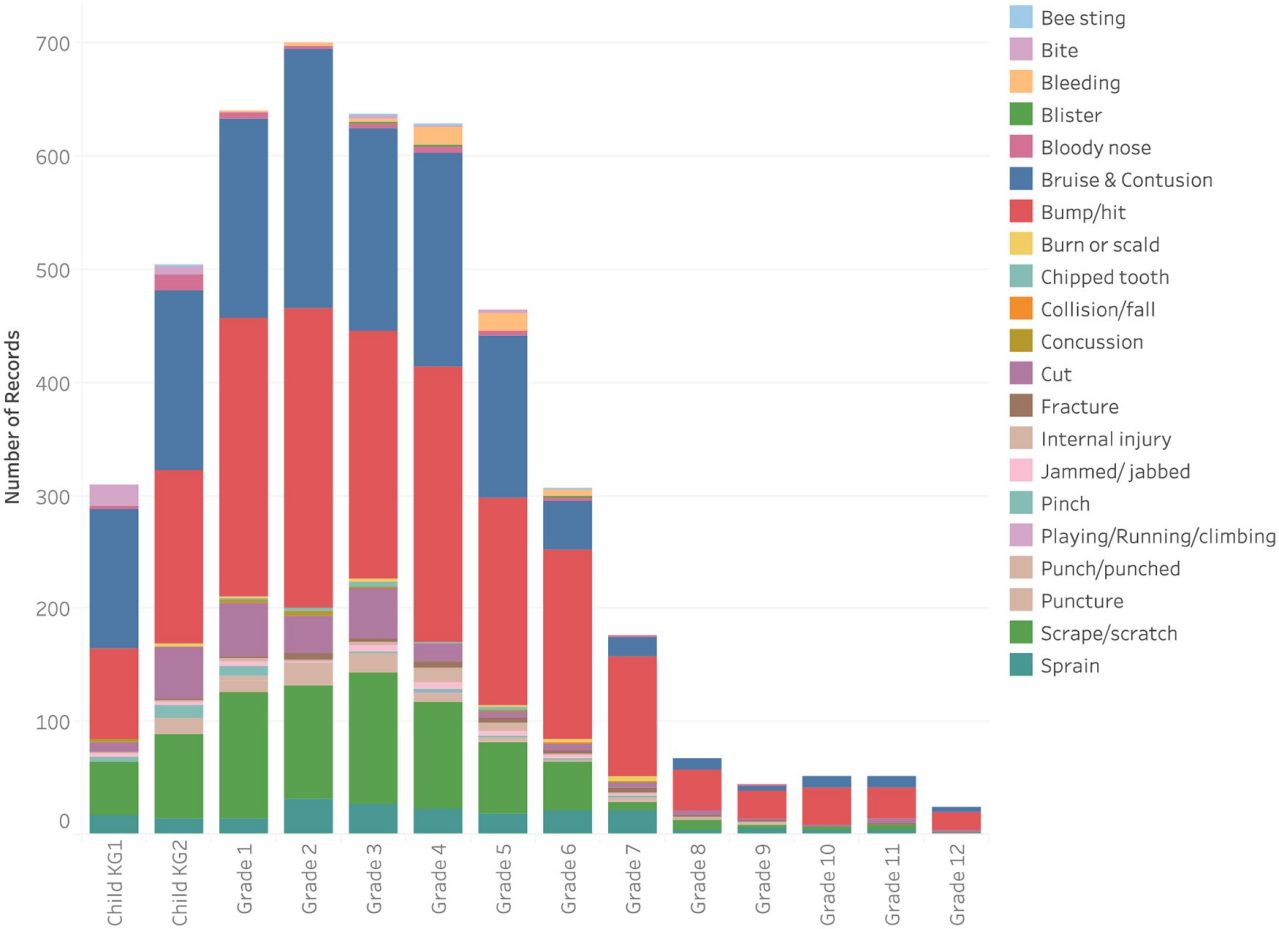
Distribution of school injury by injury type across grade levels.

The injury types with the highest rate were bumps/hits (19.3, 95% CI 16.9, 21.7), bruises (13.6, 95% CI 11.6, 15.6), scrapes (7.2, 95% CI 5.8, 8.7), cuts (2.4, 95% CI1.6, 3.3) and sprains (2.4, 95% CI 1.4, 3.1). Boys had significantly higher rates for all common injury types (p<0.0001) except for sprains. A small number of injuries resulted in fractures (n = 39). Eye injuries (n = 184) and concussions (n = 14) were less common, though when they occurred, they were treated as major injuries with the potential for repercussions on the child’s cognitive capabilities and school performance Table 2 (Fig 3).

**Table 2:** The distribution of rates of injury mechanism, nature and types between gender.

	Frequency (%)			Rate (per 1,000 students per month) (95% CI)			Gender Comparison
Variable	Boys	Girls	Total	Boys	Girls	Overall	p-value
**Injury Mechanism**							
Playing	1393 (61.5)	872 (38.5)	2265 (49.0)	29.9 (26.9, 32.9)	18.5 (16.2, 20.9)	24.2 (21.5, 26.9)	<0.0001
Running	584 (62.9)	344 (37.1)	928 (20.1)	12.5 (10.6, 14.5)	7.3 (5.8, 8.8)	9.9 (8.2, 11.6)	<0.0001
Walking	137 (54.6)	114 (45.4)	251 (5.4)	2.9 (2.0, 3.9)	2.4 (1.6, 3.3)	2.7 (1.8, 3.6)	0.1248
Other	340 (64.5)	187 (35.5)	527 (11.4)	7.3 (5.8, 8.8)	4.0 (2.8, 5.1)	5.6 (4.3, 6.9)	<0.0001
Unknown/Missing			648 (14.0)			6.9 (5.5, 8.4)	
**Intention**							
Unintentional	1612 (62.5)	966 (37.5)	2578 (55.8)	34.6 (31.4, 37.8)	20.5 (18.0, 23.0)	27.5 (24.7, 30.4)	<0.0001
Intentional	131 (72.8)	49 (27.2)	180 (3.9)	2.8 (1.9, 3.7)	1.0 (0.5, 1.6)	1.9 (1.2, 2.7)	<0.0001
Unclear	327 (63.9)	185 (36.1)	512 (11.1)	7.0 (5.6, 8.5)	3.9 (2.8, 5.0)	5.5 (4.2, 6.7)	<0.0001
Unknown/Missing			1349 (29.2)			14.4 (12.3, 16.5)	
**Injury Nature**							
Upper Extremities	730 (62.3)	441 (37.7)	1171 (25.4)	15.7 (13.5, 17.8)	9.4 (7.7, 11.0)	12.5 (10.6, 14.4)	<0.0001
Lower Extremities	779 (58.2)	560 (41.8)	1339 (29.0)	16.7 (14.5, 19.0)	11.9 (10.0, 13.8)	14.3 (12.2, 16.4)	<0.0001
Face	831 (62.3)	502 (37.7)	1333 (28.9)	17.8 (15.5, 20.1)	10.7 (8.9, 12.4)	14.2 (12.2, 16.3)	<0.0001
Head	273 (66.7)	136 (33.3)	409 (8.9)	5.9 (4.5, 7.2)	2.9 (2.0, 3.8)	4.4 (3.2, 5.5)	<0.0001
Other	190 (69.6)	83 (30.4)	273 (5.9)	4.1 (3.0, 5.2)	1.8 (1.0, 2.5)	2.9 (2.0, 3.9)	<0.0001
Unknown/Missing			94 (2.0)			1.0 (0.5, 1.6)	
**Injury Type**							
Bump/Hit	1133 (62.7)	673 (37.3)	1806 (39.1)	24.3 (21.6, 27.0)	14.3 (12.2, 16.4)	19.3 (16.9, 21.7)	<0.0001
Bruise	785 (61.7)	487 (38.3)	1272 (27.5)	16.9 (14.6, 19.1)	10.3 (8.6, 12.1)	13.6 (11.6, 15.6)	<0.0001
Scrape/Scratch	418 (61.7)	260 (38.3)	678 (14.7)	9.0 (7.3, 10.6)	5.5 (4.2, 6.8)	7.2 (5.8, 8.7)	<0.0001
Cut	151 (66.5)	76 (33.5)	227 (4.9)	3.2 (2.3, 4.2)	1.6 (0.9, 2.3)	2.4 (1.6, 3.3)	<0.0001
Sprain	115 (54.8)	95 (45.2)	210 (4.5)	2.5 (1.6, 3.3)	2.0 (1.2, 2.8)	2.2 (1.4, 3.1)	0.1455
Other	263 (62.5)	158 (37.5)	421 (9.1)	5.7 (4.3, 7.0)	3.4 (2.3, 4.4)	4.5 (3.3, 5.7)	<0.0001
Unknown/Missing			5 (0.1)			0.1 (0.0, 0.2)	
**Injury Severity**							
Minor	1130 (60.0)	753 (40.0)	1883 (40.8)	24.3 (21.6, 26.9)	16.0 (13.8, 18.2)	20.1 (17.7, 22.5)	<0.0001
Moderate	1063 (65.9)	551 (34.1)	1614 (34.9)	22.8 (20.2, 25.4)	11.7 (9.8, 13.6)	17.2 (15.0, 19.5)	<0.0001
Severe	12 (85.7)	2 (14.3)	14 (0.3)	0.3 (0.0, 0.5)	0.0 (0.0, 0.2)	0.2 (0.0, 0.4)	<0.0001
Unknown/Missing			1108 (24.0)			11.8 (9.9, 13.7)	

## Discussion

This prospective study highlights the relevance of school injuries as a common public health problem affecting the health and well-being of school-aged children in Lebanon. Key findings from this study provide a novel and relatively comprehensive understanding of the characteristics, the extent and the rates of child school injuries occurring at private schools in Lebanon. Such important knowledge is necessary to design effective school injury prevention policies and programs going forward.

Relative to other regional studies, this study estimated a school injury rate of 419/1000, which is comparable to rates suggested by Eastern Mediterranean countries, including Jordan (437/1000), Egypt (385/1000), Oman (263/1000), and UAE (307/1000) [13–21], retrieved from the Global School-Based Students Health Survey (GSBHS). Findings from this study are consistent with similar school injury studies in the region namely, that ‘Fall’ injuries are the most common mechanism, particularly among boys of a young age [13–22].

The study findings align with international literature and suggest that boys sustain more school injuries than girls of similar age groups [9,11,15]. Boys have a significantly higher injury rate for all mechanisms of injury with the exception of injuries sustained while walking, and injuries occurring in sports areas. Increased rates of injuries among boys may be explained by the fact that boys are more active than girls of the same age; they tend to display greater risk-taking behavior while underestimating potential hazards and dangers associated with their activities [27].

This study confirms that the rate and severity of injuries decrease as children age. The injury rate for elementary school children was nearly 2.4 higher than kindergarten; 2.8 higher than middle school; and, 14.5 times higher than high school. Consequently, school injury prevention measures and safety strategies should target elementary school children as ‘populations at-risk’ for effective reduction of school-based injuries.

Most injuries were unintentional, with rates about 5 times higher than ‘unclear intention’ and 12 times higher than ‘intentional injuries’. Being a male student represents a predictor of incidents of intentional injuries exhibited mainly by aggressive behavior, physical assaults, fighting, and roughness. Accordingly, there is a need to address aggressive behaviour among school children and to consider the implementation of zero-tolerance policies towards bullying and fighting that frequently result in injuries among students.

This study agrees with previous research and shows the increased risk of various types of injuries (e.g. fractures, contusions and laceration) associated with informal recreational activities and unstructured play involving playgrounds [9,11, 28–30]. Playing was the most prevalent mechanism for injury, with rates 2.5 times higher than injuries sustained while running and over 4 times higher than all other causes of injuries. The majority of the reported school-based injuries occurred during recreational hours and were linked to playground activities, predominantly during the warmer season when children were able to play outdoors. Playground injuries were common among school children; they were associated with multiple hazards and were mainly due to poor design and lack of compliance with international playground and equipment safety standards (e.g. playground slide dimension, swing height, climbing elevation, impact-absorbent ground, protective surface, equipment spacing, etc.) [31,32]. An ample number of studies have shown the strong association between the height of playground equipment and the increased risk of head- and arm-related injuries due to falls, especially with the absence of impact-absorbent playground surfacing [33–35]. Compliance with playground safety standards effectively curtails the occurrence of playground injuries and reduces risks for serious injuries, particularly among young children. Unfortunately, a limited number of schools in Lebanon have a school safety protocol in place. Hence, establishing injury prevention protocols and introducing playground safety awareness programs to educate school children about the risk of injuries are essential to the prevention of school injuries [35–37]. Moreover, the lack of active supervision during recreational hours may be associated with increased rates of injuries. Bumps/hits and bruises were almost 3 times more likely to happen compared to all other injury types. Several studies have linked adult direct supervision and active observation of children to reduced occurrences of playground injuries [38–40]. Supervisors’ presence around children can help to enforce safe behavior and allow for timely intervention in case of aggressiveness and intentionally inflicted injuries among students.

Contrary to existing literature, our study reported a low rate of sports-related injuries compared to unstructured play activities [41–43]. This may be explained by a relatively limited organized sports activity at schools in Lebanon, related to their associated setting and facility costs. Previous research has encouraged schools to invest in expanding sports curriculum and engaging students in multiple sports and outdoor activities in an effort to reduce obesity and promote an active and healthy lifestyle [44]. Organized sports and structured outdoor activities should be encouraged and adopted by schools while ensuring a safe and injury-free environment by integrating evidence-based policies and safety protocols.

This study showed that the majority of reported injuries were mainly minor or moderate in severity; severe injuries were nearly 10 times less likely to occur. Nevertheless, injuries to the face, upper extremities, and lower extremities were reported to be the most common nature of injury, approximately 3 times higher than injuries to other parts of the body. The discrepancy between the number of reported concussion cases in this study vis-a-vis the number of moderate head and face injuries raises concern, especially that the latter are not documented as serious injuries or diagnosed as mild or moderate Traumatic Brain Injury (TBI). Concussions were judged and classified as mild to moderate injury, not serious enough to warrant medical attention and follow up. This misclassification may signal a lack of adequate knowledge among school nurses on the accurate assessment of the severity of head injuries sustained, especially concussions. Specific training in the early recognition and management of concussion is encouraged, as the consequences of untreated concussion can have a significant impact on the child’s health and well-being [45,46]. Additionally, these types of child injuries should be assessed by a certified professional or specialized physician prior to the child’s return to school and play.

### Limitations

The study has some limitations to be acknowledged. The data collection was limited to private schools due to the fact that only private schools have nurses on-site to collect data and report child school injury incidents. This may affect the generalizability of the findings to the Lebanese school population as a whole. Further, given that only eleven of 74 private schools agreed to participate, there may be some limitation in generalizing the results to all private schools in Lebanon, though we have no reason to indicate that other private schools would be different from our study’s participating schools. Including only private schools in this study might also influence the reported rate of injuries as presumably private schools are relatively well-maintained with fewer overcrowded classrooms; students typically belonging to higher socioeconomic status families and more highly educated parents. In short, students in this study may be associated with multiple contributing factors linked to reduced injury morbidity and mortality rates [47–49]. Future communication with the Ministry of Health will be established to include public schools and compare findings from both private and public schools. A second limitation is related to the injury classification tool used by school nurses. Fractures and concussions were not classified as ‘severe injuries’, with the exception of a small number of cases. This may have impacted the rate and severity of the reported injuries at schools. Thirdly, not all variables were completely filled out, resulting in missing data. Most notable was that 29% of injury intent was missing, which would result in an underestimation of the injury intent rate. And lastly, this study documented child school injuries with no follow up to determine the effects of the sustained injuries in the longer term. For instance, the impact of the injury on children missing days of schools or any short or long-term consequences on students’ physical and/or academic performance.

## Conclusions

This is a first-time study in Lebanon that quantifies child school injury rates, investigates injury types and mechanisms, and highlights their direct associated risk factors. Examining school-based injuries and their associated behavior and risk factors is crucial to designing and adopting effective injury prevention measures and strategies at schools in the future.

As part of growing older, children’s risky behavior varies, resulting in various types of injuries associated with children’s age, sex and their surrounding environment. In an attempt to curtail child injuries, there is a need to identify the mechanisms and underlying factors associated with these injuries. By recognizing, changing and controlling these factors, most children’s injuries can be prevented. Further study is needed to investigate the repercussion of moderate to severe injuries on a child’s health and academic performance.

### Implications for school health

Findings from this study will be shared with the Ministry of Education and will call for the development and implementation of school safety protocols and strategies that align with international safety standards, mainly playground equipment safety. Promoting a safe and injury-free school environment is critical for the physical, emotional, and cognitive development and well-being of children. School administrators, teachers and nurses should mobilize the efforts to ensure child safety and reduce school-based injuries.

Moreover, this study calls for the implementation of a school-based injury surveillance tool and system that will serve as a platform to provide the evidence needed to design effective and strategic injury prevention programs and interventions at schools.

The study evidence will be utilized to design and develop educational training courses that will be offered to school nurses, administrators and coaches to impart necessary skills and adequate training on the latest injury prevention theories and best practices to effectively diagnose and manage severe school injuries, particularly traumatic brain injuries (concussions).

## Supporting information

S1 Data (XLSX)
